# Bioactive potential of the wild mushroom *Astraeus hygrometricus* in South-west India

**DOI:** 10.1080/21501203.2016.1260663

**Published:** 2016-11-28

**Authors:** Mundamoole Pavithra, Kandikere R. Sridhar, Ammatanda A. Greeshma, Kaori Tomita-Yokotani

**Affiliations:** a Department of Biosciences, Mangalore University, Mangalore, India; b Applied Biochemistry and Life Sciences, University of Tsukuba, Tsukuba, Japan

**Keywords:** Antioxidant, food security, ectomycorrhizae, gasteromycete, nutraceutical value, tribes

## Abstract

The wild mushroom *Astraeus hygrometricus* is a traditional delicacy during the monsoon season in South-western India. Bioactive principles and antioxidant potential of uncooked and pressure-cooked tender mushroom have been evaluated. Seven bioactive principles of mushroom (tannins, flavonoids, vitamin C, phytic acid, lycopene, β-carotene and trypsin inhibition) were significantly higher, while total phenolics content was significantly lower in uncooked than in cooked samples. Mushroom was devoid of L-DOPA, whereas only uncooked samples showed haemagglutinin activity against A^+^ blood group. The principal component analysis of uncooked mushroom showed only two bioactive principles clustered with two antioxidant properties, while in cooked mushroom five bioactive principles clustered with three antioxidant properties depicting the nutraceutical potential of cooked mushroom. Future studies should focus on appropriate thermal treatment, which retain maximum bioactive and antioxidant potential to combat health- and lifestyle-related ailments. The *A. hygrometricus* is ectomycorrhizal, conservation of its host tree species is utmost importance in improvement and expansion of its yield to sustain food security and economic gains of local tribals.

## 1. Introduction

Mushrooms are historically known as integral part of human nutrition and health as alternative to plant- and animal-derived products (Boa 2004; Oboh and Shodehinde 2009). Edible mushrooms are also known to meet the protein-energy requirement due to their proximal qualities, low calorie, minerals, vitamins, essential amino acids and essential fatty acids (Murcia et al. 2002; Sadler 2003; Sanmee et al. 2003; Agrahar-Murugkar and Subbulakshmi 2005; Sudheep and Sridhar 2014; Paterson and Lima 2015). Despite commercially cultivated mushrooms, a wide variety of wild mushrooms is consumed by the native or tribal sect worldwide (Boa 2004). The Western Ghats and west coast of India are known for several nutritionally and medicinally versatile wild mushrooms (e.g. Pahlevanlo and Janardhana 2012; Karun and Sridhar 2014, 2016a; Pavithra et al. 2015, 2016). Some of the commonly used wild mushrooms by the native people in the Western Ghats and west coast of India include: *Agaricus* spp., *Amanita* spp., *Astreaus* spp., *Auriculria* spp., *Boletus* spp., *Lentinus* spp., *Pleurotus* spp. and *Termitomyces* spp.

The gasteromycete edible mushroom *Astraeus hygrometricus* is ectomycorrhizal with several tree species in the South-west India (e.g. *Acacia auriculiformis, Anacardium occidentale, Artocarpus hirsutus, Holigarna arnottiana, Hopea parviflora, H. ponga, Phyllanthus emblica* and *Syzygium cumini*) (Pavithra et al. 2015). It is known for several medicinal properties like immunoenhancement, splenocyte activation, cardioprotection, hepatoprotection, hypoglycaemic, anti-inflammatory, free radical-scavenger, lipid peroxidation inhibition, anticandidal and anti-leishmanial activities (Chakraborty et al. 2004; Biswas et al. 2010a, 2010b, 2011a, 2011b, 2012; Lai et al. 2012; Biswas and Acharya 2013; Mandal et al. 2015; Shameem et al. 2015). As *A. hygrometricus* is one of the widely consumed mushrooms in the South-west India, the aim of this study is to project its nutraceutical properties in uncooked and cooked state by assessing bioactive principles and antioxidant properties.

## 2. Materials and methods

### 2.1. Mushroom sampling and processing

**Figure 1.**
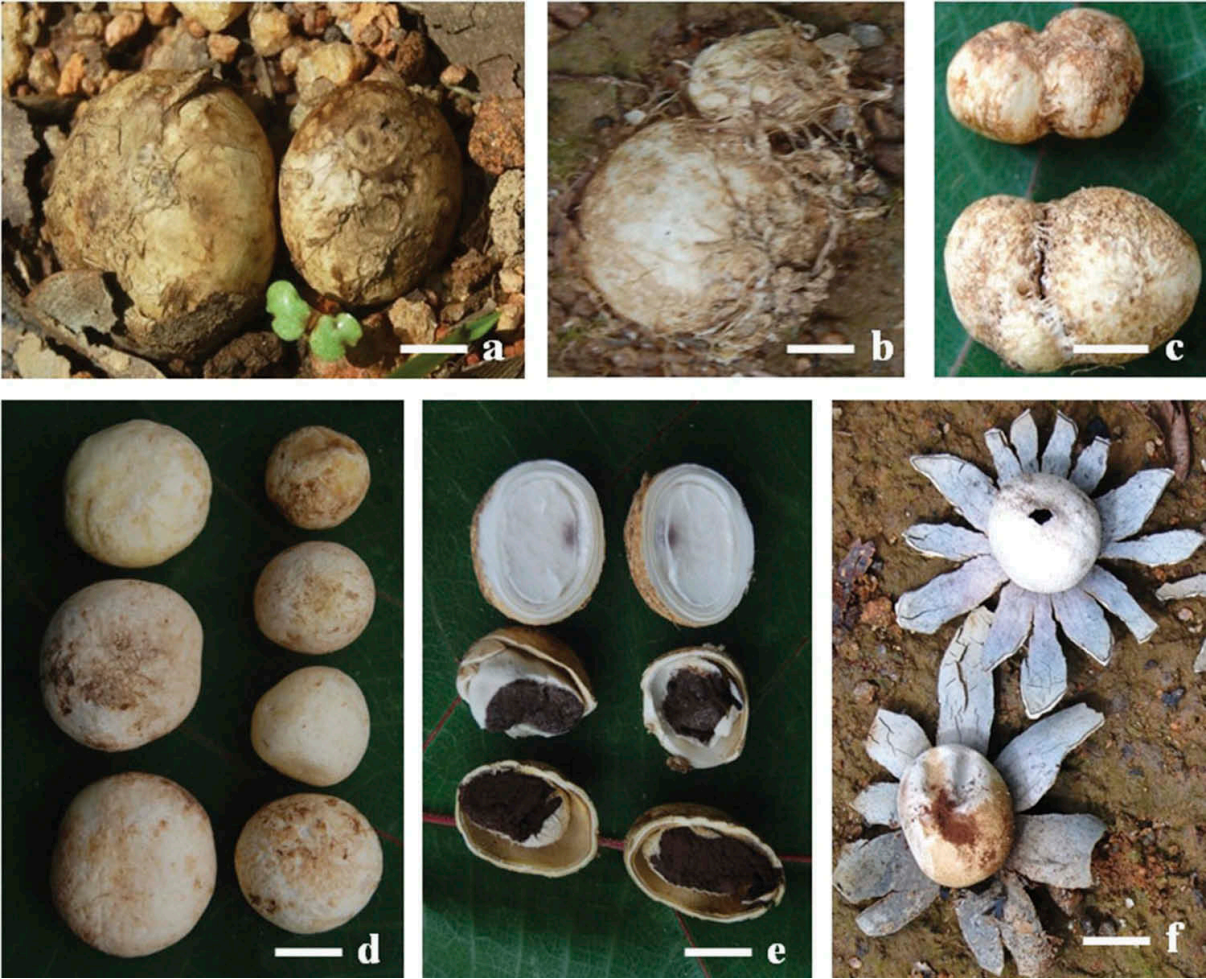
Partially matured (a) and immature (b) fruit bodies of *Astraeus hygrometricus* embedded in lateritic soil (note fruit bodies partially covered by roots in b), fused two fruit bodies (c), cleaned tender fruit bodies (d), cut open fruit bodies (e) (note edible tender immature fruit body with white interior in top used for analysis and mature fruit bodies blackened interior were discarded) and mature and near spent stage of mushroom (f).

Tender epigeous *A*. *hygrometricus* (Pers.) Morgan were collected from the Karkala forests of foothill regions of the Western Ghats of South-west India (13°12′N, 74°58′E; 65–90 m asl) during June and July 2015. During tender stage, they are edible, having spherical to oval shape with bone-white surface (Figure 1). About 250–500 g tender mushrooms were collected in polythene bags from five different locations of ~50 m apart as replicates. They were brought to the laboratory in cool pack within 3–4 h, soaked in water and slightly scrubbed to remove adhered soil and mycelial mass followed by blotting. Each replicate has been divided into two groups, fruit bodies were cut into half to ascertain tenderness (white flesh) and to discard matured ones (black interior) (Figure 1(e)). The first group of five replicates served as uncooked samples was oven dried (50–55°C) until the moisture reduced below 10%. The second group was pressure-cooked with distilled water (1:1 v/v) in a household cooker (Deluxe stainless steel, TTK Prestige™, Prestige Ltd., Hyderabad, India; capacity, 6.5 l) followed by oven drying. Dried uncooked and cooked samples were milled in Wiley mill (mesh #30) and refrigerated (4°C) in airtight containers for analysis.

### 2.2. Bioactive components

#### 2.2.1. Phenolics

Method outlined by Rosset et al. (1982) was followed to assess the total phenolics content of mushroom flours. Samples (100 mg) were extracted with methanol (50%; 5 ml) in water bath (95 ± 1°C; 10 min) and centrifuged (1500 rpm) to collect the supernatant. Extraction was repeated for the pellet, supernatant was pooled to make up to 10 ml. Extract aliquots (0.5 ml) were mixed with distilled water (0.5 ml) followed by mixing with Na_2_CO_3_ (2% in 0.1 N NaOH) (5 ml) and incubated at room temperature (10 min). The Folin–Ciocalteu’s reagent (1:2 v/v dilution with distilled water) (0.5 ml) was added and read the absorbance (725 nm) (UV-VIS Spectrophotometer-118; Systronics, Ahmedabad, Gujarat, India) using tannic acid as standard to express total phenolics in mg of tannic acid equivalents per gram flour (mg TAEs g^−1^).

#### 2.2.2. Tannins

Vanillin-HCl method followed by Burns (1971) was employed to assess tannins in mushroom flours. The flour (1 g) was extracted with methanol (50 ml) at room temperature (24 h), centrifuged (1500 rpm) to collect the supernatant. Aliquots (1 ml) were treated with vanillin hydrochloride reagent (4% vanillin in methanol + 8% concentrated HCl in methanol; 1:1) (5 ml), incubated at room temperature (20 min), read absorbance of developed colour (500 nm) using catechin (98% High Performance Liquid Chromatography (HPLC) grade; Sigma Aldrich, USA) as standard to express tannins in mg of catechin equivalents per gram flour (mg CEs g^−1^).

#### 2.2.3. Flavonoids

Procedure outlined by Chang et al. (2002) was adapted to assess flavonoids in mushroom flours. The flour was extracted in methanol (1 mg ml^−1^), aliquots of methanol extract (0.5 ml) were mixed with methanol (1.5 ml), AlCl_3_ (10%) (0.1 ml), KCH_3_CO_2_ (1 M) (0.1 ml) and distilled water (2.8 ml). The mixture was incubated at room temperature (30 min) and absorbance was read (415 nm) using quercetin as standard to express flavonoids in mg of quercetin equivalents per gram flour (mg QEs g^−1^).

#### 2.2.4. Vitamin C

Method outlined by Roe (1954) was employed with a slight modification to determine vitamin C in mushroom flours. The flour (1 g) was extracted with trichloroacetic acid (TCA) (5%) (10 ml), aliquots (0.2 ml) were made up to 1 ml using TCA (5%) and addition of chromogen (0.6% CuSO_4_, 5 ml + 5% of CH_4_N_2_S, 5 ml + 2.2% of 2,4-dinitrophenylhydrazine, 90 ml) (1 ml). The mixture was boiled (10 min), cooled to room temperature, H_2_SO_4_ was added (65%) (4 ml), incubated at room temperature (30 min) and absorbance was measured (540 nm) using ascorbic acid as standard to express vitamin C content in mg of ascorbic acid equivalents per gram flour (mg AAEs g^−1^).

#### 2.2.5. Phytic acid

To estimate phytic acid in mushroom flours, procedures advocated by Deshpande et al. (1982) and Sathe et al. (1983) were followed with a slight modification. The flour (1 g) was extracted with HCl (1.2%) containing Na_2_SO_4_ (10%) (10 ml) at room temperature (2 h), centrifuged and supernatant was made up to 10 ml. Phytate phosphorous was estimated before and after precipitation of phytic acid by FeCl_3_. The extract (5 ml) mixed with FeCl_3_ solution (FeCl_3_, 2 g + concentrated HCl, 16.3 ml; diluted to 1 l) (3 ml) was added, vortexed, boiled on a water bath (75 min), allowed to cool at room temperature (1 h) before centrifuge (2000 × g) (10 min) and filtered (Whatman #1). The supernatant was made up to 10 ml with distilled water, soluble phosphorous was assessed according to vanadomolybdophosphoric acid method (AOAC 1995) and absorbance was measured after 30 min (460 nm) to express phytic acid content per cent (mg 100 mg^−1^):

(1) $$Phytic\;acid\;\left(\%\right)=\left(A_{460}\times 28.18\right)÷100,$$

(where A = absorbance)

#### 2.2.6. Carotenoids

The carotenoids lycopene and β-Carotene contents of mushroom extract were assayed based on method by Nagata and Yamashita (1992) with a slight modification. Mushroom flour (5 g) was extracted in methanol (100 ml) at room temperature (24 h), filtered (Whatman #4), extraction was repeated and pooled extract was evaporated (42°C) to dryness. Dried methanol extract of mushrooms (100 mg) was dissolved by vortexing (1 min) in mixture of acetone + hexane (4:6 v/v) (10 ml) and filtered (Whatman #4). The absorbance of filtrate was measured at different wavelengths (453, 505 and 663 nm) to calculate lycopene and β-Carotene contents in 100 ml extract to express their concentration in mg per gram (mg g^−1^).

(2) $$\begin{array}{l} \text{Lycopene}\;\left(\text{mg}\;\text{100}\;\;{\text{ml}}^{\text{-1}}\right)\\ \;\;\;\;\;\;\;\;\;\;\text{=}(\text{-0}.\text{0458}\;\text{A}{\;}_{\text{6630}}\text{+0}.\text{372}\;\text{A}{\;}_{\text{505}}\text{-0}.\text{0806A}{\;}_{\text{453}}) \end{array}$$

(3) $$\begin{array}{l} \beta \text{-carotene}\;\left(\text{mg}\;{\text{100ml}}^{\text{-1}}\right)\\ \;\;\;\;\;\;\;\;\;\;\;\;\text{=}(\text{0}.\text{216}\;\text{A}{\;}_{\text{663}}\text{-0}.\text{304A}{\;}_{\text{505}}\;\text{+}\;\text{0}.\text{452A}\;{\;}_{\text{453}}) \end{array}$$

#### 2.2.7. L- DOPA

The L-3,4-Dihydroxyphenylalanine (L-DOPA) of mushroom samples was determined based on the method by Fujii et al. (1991). Samples of mushroom flour was mixed with distilled water (1 ml) followed by incubation at room temperature (2 h), centrifuged (1500 rpm) and supernatant was concentrated to dryness using rotary evaporator. The dried extract was dissolved in distilled water, filtered (Ultrafilter; Toyo Roshi Kaisha Ltd., Japan) and incubated at room temperature (overnight) to eliminate higher molecular weight compounds. The low molecular weight fraction was purified using a octadecyl-silica mini column (C18 Sep-Pak Cartridge; Waters) with water (100%). On concentrating the extract to dryness, L-DOPA was determined using HPLC (Tosoh system DP-8020; UV-8020, 280 nm; Column, Aqua Mightsil; Kanto chemical Co. Inc., Japan) and LC-ESI/MS (Positive mode; Waters Associates Inc., Milford, MA).

#### 2.2.8. Trypsin inhibition activity

Trypsin inhibition activity of mushroom flours, enzymatic protocol proposed by Kakade et al. (1974) was employed with a slight modification (Liu and Markakis 1989). Mushroom flour (1 g) was extracted with NaOH (0.01 N) (50 ml), the extract (1 ml) was made up to 2 ml in distilled water. Trypsin solution (4 mg in 200 ml 0.001 M HCl) (2 ml) was added, incubated in a water bath (37°C) (10 min). To each sample, BAPNA (40 mg of N-a-Benzoyl-DL-Arginine p-nitroanilide hydrochloride in 1 ml C_2_H_6_OS diluted to 100 ml with Tris buffer at 37°C) was added (5 ml), incubated (10 min), the reaction was terminated by addition of acetic acid (30%) (1 ml). On thorough vortexing, the mixture was filtered and absorbance was measured (410 nm) using reagent blank (distilled water) and control without extract (1 ml of distilled water + 2 ml each of distilled water and trypsin + 5 ml BAPNA + 1 ml of 30% acetic acid). The trypsin inhibition (TIu mg^−1^) is defined as release of p-nitroanilide (1 μM min^−1^) by the enzyme:

(4) $$\begin{array}{l} \text{TIu}\;{\text{mg}}^{-1}\text{=}[\{({\text{A}}_{\text{c410}}{\text{-A}}_{\text{s410}})\times \text{100})\}\\ \;\;\;\;\;\;\;\;\;\;\;\;\;\;\;\;\;\;\;\;\;\;\;\;{\text{ml}}^{\text{-1}}\text{diluted}\;\text{mushroom}\;\text{extract}]\\ \;\;÷\left[\left(\text{mg}\;\text{sample}\;{\text{ml}}^{\text{-1}}\text{diluted}\;\text{mushroom}\;\text{extract}\right)\right], \end{array}$$

(4)

(where A_c_, absorbance of the control; A_s_, absorbance of sample).

#### 2.2.9. Haemagglutinin activity

The method proposed by Occenã et al. (2007) was adapted to determine haemagglutinin activity of mushroom flours with a slight modification. Sample of mushroom flour (2 g) was suspended in NaCl (0.9%) (20 ml), shaken vigorously, allowed to stand (1 h), centrifuged (2000 g) (10 min) to get clear solution, filtered and filtrate was considered as crude agglutinin extract. Human RBCs were separated from different blood groups (A^+^, B^+^, AB^+^ and O^+^) (5 ml), centrifuged (2000 g) (10 min), diluted with cold saline (0.9%) (1:4) and centrifuged (2000 g) (10 min) to discard the supernatant. The pellet of RBC was washed by saline till the supernatant became colourless. Washed erythrocytes (4 ml) were suspended in phosphate buffer (0.0006 M; pH 7.4) (100 ml), trypsin solution (2%) (1 ml) was added to washed erythrocytes (10 ml), mixed and incubated in water bath (37°C) (1 h). The trypsinised erythrocytes were washed (4–5 times in saline) to eliminate traces of trypsin and packed cells (1.2–1.5 ml) were suspended in saline (100 ml). In microtiter plates (12 wells × 8 rows), the phosphate buffer (50 µl) was transferred to the well #1–12 (the well #12 with plain phosphate buffer served as control). The crude agglutinin extract (50 µl) was dispensed to well #1, mixed, followed by serial dilution up to well #11 and trypsinised human RBC (in saline) (50 μl) was dispensed to well #1–12. On mixing the contents were incubated at room temperature (4 h). The haemagglutination in each well was observed and haemagglutinating unit per gram (Hu g^−1^) was determined:

(5) $$Hug^{-1}=\left(D_{a}\times D_{b}\times S\right)÷V,$$

(where Da, dilution factor of extract in well #1, it remains as 1 if the original extract is not diluted; Db, dilution factor of well containing 1 Hu is the well in which haemagglutination was first seen; S, ml original extract per gram mushroom flour; V, volume of extract in well #1).

### 2.3. Antioxidant potential

The antioxidant potential of mushrooms was dependent on different components and demands to evaluate at least two methods for fair assessment (Wong et al. 2006). Antioxidant potential of *A. hygrometricus* was assessed based on four assay methods: total antioxidant activity (TAA); Fe^2+^ ion-chelating capacity; DPPH radical-scavenging activity; reducing power.

Samples of mushroom flour (0.5 g) were extracted by methanol (30 ml) on a rotary shaker (150 rpm) (48 h). The extract was centrifuged to collect supernatant in a pre-weighed Petri plate and allowed to dry at room temperature. The mass of extract was assessed gravimetrically followed by dissolving in known quantity of methanol (1 mg ml^−1^) to evaluate antioxidant potential.

#### 2.3.1. Total antioxidant activity

The TAA was assessed according to the method by Prieto et al. (1999). Mushroom extract (0.1 ml) was mixed with a reagent mixture (H_2_SO_4_, 0.6 M + Na_2_SO_4_, 28 mM + NH_4_Mo_7_O_24_.2H_2_O, 4 mM) (1 ml), incubated (95°C) (90 min), cooled and absorbance of phosphomolybdenum complex was measured (695 nm) with methanol blank using ascorbic acid as standard to express TAA in μM equivalent to ascorbic acid per gram of flour (μM AAEs g^−1^).

#### 2.3.2. Ferrous ion-chelating capacity

The Fe^2^^+^-chelating capacity was determined by following method by Hsu et al. (2003). To mushroom extract, (1 ml) FeCl_2_ (2 mM) (0.1 ml) + ferrozine (5 mM) (0.2 ml) were added and made up to 5 ml in methanol. On incubation at room temperature (10 min), absorbance of Fe^2^^+^–ferrozine complex formed was measured (562 nm), the sample without extract served as control and Fe^2^^+^-chelating capacity was calculated:

(6) $$Fe^{2+}chelating\;capacity\;\left(\%\right)=1-(A_{s562}÷A_{c562})\times 100,$$

(where A_c_, absorbance of the control; A_s_, absorbance of sample).

#### 2.3.3. Reducing power

The method outlined by Oyaizu (1986) was followed with a minor modification to determine reducing power of the mushroom extract. Extract concentrations (0.2–1 mg) (0.2–1 ml stock) were mixed with phosphate buffer (0.2 M, pH 6.6) (2.5 ml) and K_3_Fe(CN)_6_ (1%) (2.5 ml). The mixture was incubated (50°C) (20 min), TCA (10%) (2.5 ml) was added, centrifuged (3000 rpm) (10 min) and supernatant (2.5 ml) was mixed with distilled water (2.5 ml). To the mixture, FeCl_3_ (0.1%) (0.5 ml) was added to measure absorbance (700 nm) to ascertain increase in absorbance as indication of increased reducing power.

#### 2.3.4. DPPH radical-scavenging activity

To determine free radical-scavenging activity, the method by Singh et al. (2002) was employed. Extract concentrations (0.2–1 mg) (0.2–1 ml stock) were made up to 1 ml using methanol, the DPPH (0.01 mM) (4 ml) was added and allowed to react at room temperature (20 min). Reagents without extract served as control, the absorbance of mixture was measured (517 nm) and free radical-scavenging activity was calculated:

(7) $$\begin{array}{l} \text{Free}\;\text{radical}\;\text{-scavenging}\;\text{activity}\;\left(\%\right)\\ \;\;\;\;\;\;\;\;\;\;\text{=}[({\text{A}}_{\text{c517}}{\text{-A}}_{\text{s517}})÷\left({\text{A}}_{\text{c517}}\right)]\times \text{100}, \end{array}$$

(where Ac, absorbance of the control; As, is absorbance of sample).

### 2.4. Data analysis

The differences in quantity of bioactive components between uncooked and cooked mushroom flours were assessed by Student *t*-test (Statistica version #8.0) (StatSoft 2008). The principal component analysis (PCA) was employed to find out relationship between bioactive components of mushroom (total phenolics, tannins, flavonoids, vitamin C, phytic acid, lycopene, β-carotene and trypsin inhibition) and antioxidant potential (total antioxidant activity, Fe^2^^+^-chelating capacity, reducing power and DPPH radical-scavenging activity) of uncooked and cooked mushroom (SPSS 16.0: www.spss.com). The PCA score plots of uncooked and cooked mushroom samples were grouped among bioactive components and antioxidant potential.

## 3. Results and discussion

Nutritional and health-promoting potential of wild mushrooms are of special interest as they are different from plant and animal sources. Bioactive potential of wild mushrooms are increasingly recognised owing to a wide range of properties like radical-scavenging, antimicrobial, anticancer, antidiabetes, hepatoprotective, cosmeceutical and nutricosmetic potentials (Lindequist et al. 2005; Biswas et al. 2011b; Kalogeropoulos et al. 2013; Shameem et al. 2015; Smith et al. 2015; Taofiq et al. 2016). Recently, mushroom-based antioxidants have attracted wide attention worldwide against synthetic antioxidants (e.g. butylated hydroxytoluene, butylated hydroxyanisole and tertiary butyl hydroquinone). Moreover, there is a stringent control of use of synthetic antioxidants in food industry as they are known to be carcinogenic (Botterweck et al. 2000; Ren et al. 2014).

### 3.1. Bioactive components

**Figure 2.**
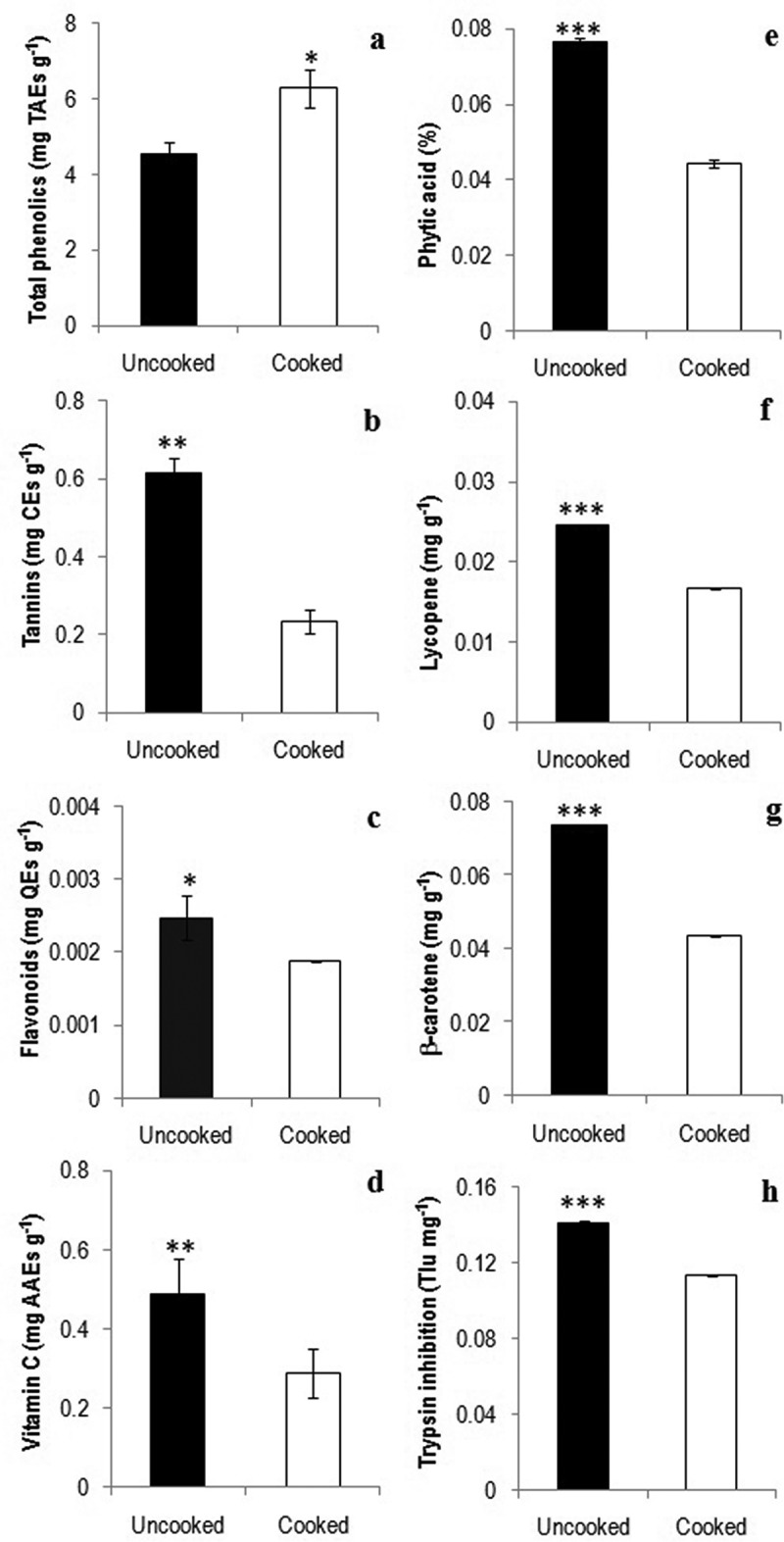
Bioactive principles of tender fruit bodies of *Astraeus hygrometricus*: total phenolics (a), tannins (b), flavonoids (c), vitamin C (d), phytic acid (e), lycopene (f), β-carotene (g) and trypsin inhibition (h) (*t*-test: *, *p *< 0.05; **, *p* < 0.01; ***, *p* < 0.001).

Consumption of natural produce with substantial amount of phenolic compounds serve as primary free radical terminators leading to reduce of incidence cancer, heart diseases and atherosclerosis (Randhir et al. 2008; Alothman et al. 2009). The antioxidant potential of many wild mushrooms in Asia has been correlated with total phenolics (Cheung and Cheung 2005; Lo and Cheung 2005). Interestingly, pressure-cooking resulted in significant increase in total phenolics (*p* < 0.05) in *A. hygrometricus*, while it was opposite for tannins (*p* < 0.01) (Figure 2(a,b)). It is likely pressure-cooking leads to increased extractactability of phenolics in *A. hygrometricus*. Unlike *A. hygrometricus*, in *Termitomyces umkowaan* total phenolics was substantially higher in uncooked samples, but pressure-cooking significantly decreased almost close to its content in *A. hygrometricus* (Karun et al. 2016b). The tannin content of uncooked and cooked *A. hygrometricus* was higher compared with *T. umkowaan* (Karun et al. 2016b). As total phenolics (4.25 vs. 1.4%) as well as tannins (1.14 vs. 1.1%) of inner part of fruit body of *A. hygrometricus* collected from Central India was higher than outer part (Singh 2011) shows their differential distribution in edible fruit bodies.

Although flavonoids are biologically versatile compounds well known for their positive impact on human health (antidiabetic, anti-inflammatory, hepatoprotective and cardioprotective properties (Champ 2002; Tapas et al. 2008), studies on mushrooms are scanty (Barros et al. 2008; Gursoy et al. 2009). Flavonoids significantly decreased in cooked *A. hygrometricus* (*p* < 0.05) (Figure 2(c)), however, its content was substantially lower than uncooked and cooked *T. umkowaan* (Karun et al. 2016b). The potent antioxidant vitamin C in foods is known to decrease due to thermal treatment (Gregory 1996; Podmore et al. 1998). Pressure-cooking significantly decreased vitamin C in *A. hygrometricus* (*p* < 0.01) (Figure 2(d)), but its content in uncooked as well as cooked samples was substantially higher than *T. umkowaan* (Karun et al. 2016b). Phytic acid is a common antioxidant in foods and serves as antineoplastic agent and also responsible for many benefits (e.g. postprandial glucose absorption, reduction of renal lithiasis and prevention of dental caries) (Kumar et al. 2010). The quantity of phytic acid was meagre in uncooked *A. hygrometricus*, it decreased significantly on cooking (*p* < 0.001) (Figure 2(e)). Interestingly, low amount of phytic acid in food stuffs is beneficial in augmenting bioavailability of proteins and minerals (by lowering phytate-protein and phytate-protein–mineral complexes) (Siddhuraju and Becker 2001).

As in vegetables, carotenoids are also important natural antioxidants in mushrooms. The antioxidant potential of three wild edible mushrooms in Portugal has been correlated with quantity of carotenoids (Barros et al. 2007). Lycopene as well as β-carotene were considerably decreased on cooking *A. hygrometricus* (*p* < 0.001) (Figure 2(f,g)), but irrespective of processing their quantity were higher than three wild edible mushrooms in Portugal (Barros et al. 2007) and two wild edible mushrooms of North-east India (Mitra et al. 2014, 2015).

Being non-protein amino acid, L-DOPA facilitates in treating Parkinson’s disease (Hornykiewicz 2002). Unlike *T. umkowaan* (Karun et al. 2016b), L-DOPA was absent in uncooked as well as cooked *A. hygrometricus*. Trypsin inhibitors in food result in utilising sulphur amino acids for trypsin and chymotrypsin synthesis leading to deficiency of sulphur amino acids (Liener and Kakade 1980). As the quantity of trypsin inhibitors was low in uncooked *A. hygrometricus*, which further decreased on cooking (*p *< 0.001) (Figure 2(h)) may not substantially influence the nutritional quality. Lectins in food stuffs serve as immunomodulators and responsible for hemagglutinin activity (Hartmann and Meisel 2007); however, its absence in food stuffs will be nutritionally beneficial. There was no *in vitro* hemagglutinin activity by *A. hygrometricus* against blood groups (A^+^, B^+^, O^+^ and AB^+^) except for uncooked samples against group A^+^ (200 Hu g^−1^) (Table 1) denoting nutritional value addition of cooked *A. hygrometricus*.

**Table 1.** Haemagglutinin activity (Hu g^−1^) of uncooked and cooked tender *Astraeus hygrometricus* against human blood groups.

Treatment	Blood group			
	A^+^	B^+^	AB^+^	O^+^
Uncooked	200	0	0	0
Cooked	0	0	0	0

### 3.2. Antioxidant potential

**Figure 3.**
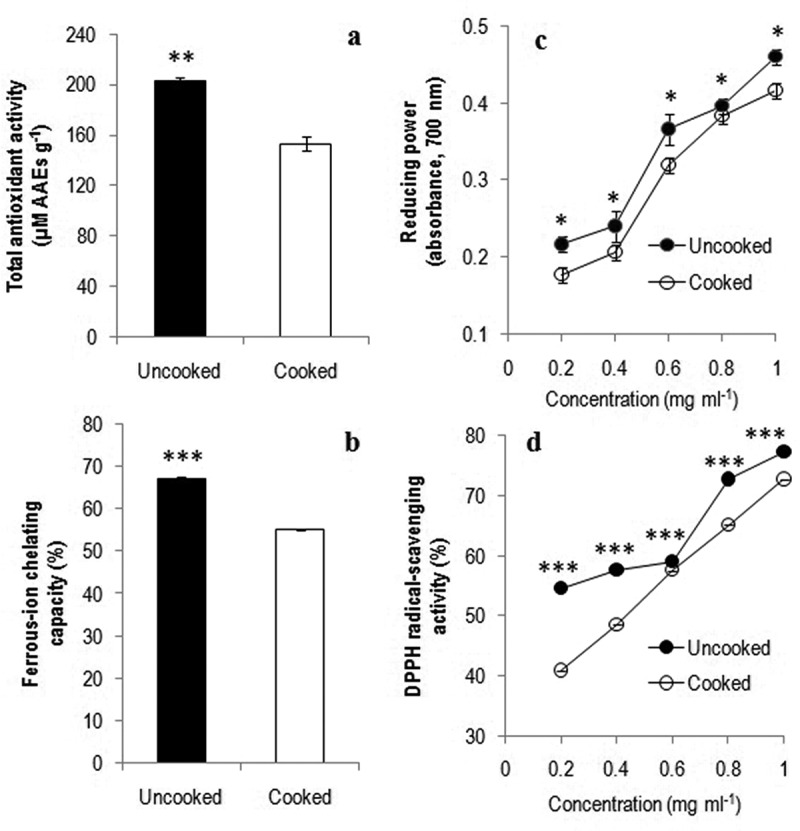
Antioxidant activities of tender fruit bodies of *Astraeus hygrometricus*: total antioxidant activity (a), ferrous ion-chelating capacity (b), reducing power (c) and DPPH radical-scavenging activity (d) (*t*-test: *, *p *< 0.05; **, *p* < 0.01; ***, *p* < 0.001).

The antioxidant potential of mushrooms is influenced by various bioactive compounds. The total antioxidant activity was significantly decreased up to one-third in cooked *A. hygrometricus* (*p* < 0.01) (Figure 3(a)), which is lower than *T. umkowaan* (Karun et al. 2016b). Such results might be due to polysaccharides present in *A. hygrometricus* as depicted by Zhang et al. (2011). Lipid peroxidation due to catalytic activity of metal-ions is responsible for deterioration of food stuffs leading to cause arthritis and cancer (Gordon 1990; Halliwell et al. 1995). The pro-oxidant metals will be converted into stable compounds by metal chelators, which are responsible to reduce the damaging effect (Leopoldini et al. 2006; Soares et al. 2009). In *A. hygrometricus*, although cooking decreased Fe^+^ chelating activity (*p* < 0.001) (Figure 3(b)), it was not decreased too much and hence chelating agents serve as secondary antioxidants leading to reduce the redox capacity of metal ions. Reducing power and DPPH radical-scavenging properties are widely used assays to ascertain the antioxidant capacity of bioactive components. The reducing activity of *A. hygrometricus* was concentration dependent, which did not decrease too much on cooking (*p* < 0.05) (Figure 3(c)); however, it is substantially higher than *T. umkowaan* (1 mg ml^−1^) (Karun et al. 2016b). The DPPH radical-scavenging activity also followed the same trend like reducing power (*p* < 0.001) (Figure 3(d)) and it was several folds higher than *T. umkowaan* (1 mg ml^−1^) (Karun et al. 2016b).

### 3.3. Bioactive components vs. antioxidant potential

**Figure 4.**
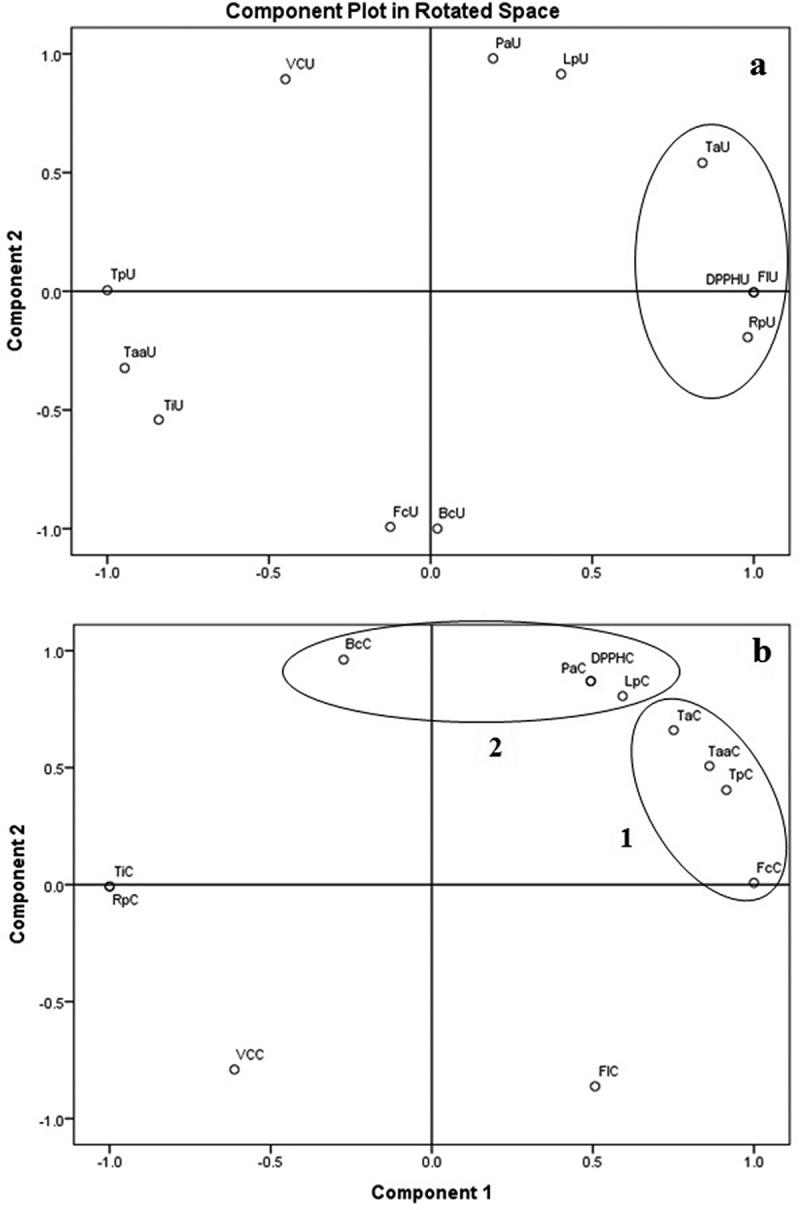
Principle component analysis of uncooked (with suffix U) (a) and cooked (with suffix C) (b) tender fruit bodies of *Astraeus hygrometricus*: [(Bioactive principles: total phenolics (Tp), tannins (Ta), flavonoids (Fl) and vitamin C (VC), phytic acid (Pa), lycopene (Lp), β-carotene (Bc) and trypsin inhibition (Ti); antioxidant activities: total antioxidant activity (Taa), ferrous ion-chelating capacity (Fc), reducing power assay (Rp) and DPPH radical-scavenging activity (DPPH)].

The PCA of bioactive principles in uncooked *A. hygrometricus* against antioxidant potential resulted in two components (Eigen value, <1), which accounted for 100% variance. The rotated score plot revealed 62.07% variance for component 1, while 37.93% for component 2 (Figure 4(a)). The tannins (TaU) and flavonoids (FlU) were clustered with reducing power (RpU) and DPPH radical-scavenging activity (DPPHU) in the right side of score plot (Figure 4(a)). The PCA of bioactive principles of cooked *A. hygrometricus* against antioxidant potential resulted in two components (Eigen value, <1), which accounted for 100% variance. The rotated score plot revealed 71.17 % variance for component 1, while 28.83 % for component 2 (Figure 4(b)). Five bioactive principles of cooked mushroom (total phenolics, TpC; tannins, TaC; phytic acid, PaC; β-Carotene, BcC and lycopene, LpC) were clustered with three antioxidant properties (total antioxidant activity, TaaC; Fe^2^^+^-chelating capacity, FcC and DPPH radical-scavenging activity, DPPHC) in two groups in the right side of score plot (Figures 4(b), 1 and 2). The result of PCA between bioactive principles and antioxidant properties showed different pictures between uncooked and cooked mushroom. In uncooked mushroom, only two bioactive principles were clustered with two antioxidant potential, while in cooked mushroom five bioactive principles clustered with three antioxidant potential depicting the nutraceutical value of cooked mushroom.

### 3.4. Outlook

Compared to the plant-based foods, mushrooms are relatively underexplored source of nutraceuticals. Uncooked and cooked tender *A. hygrometricus* as edible wild mushroom of the South-western India endowed with several bioactive principles and potential antioxidant properties serve as important nutraceutical source. Evaluation of nutritional, functional and physical properties of *A. hygrometricus* is necessary for in depth knowledge on their nutraceutical and industrial applications as functional foods. Further progress is needed especially establishing suitable methods of cooking (e.g. partial, extrusion and microwave) to retain high nutraceutical potential in *A. hygrometricus* to combat specific ailment. However, uncooked or processed mushroom may serve the challenges in prevention of health-related and lifestyle-dependent human diseases in future. Conservation of *A. hygrometricus* and its ecosystem is utmost important in order to support natives or tribals those dependent on such resource for their income up to 3 months during wet season in the Western Ghats and west coast. There are several locations where *A. hygrometricus* and allied species have been identified in South-western India (Pavithra et al. 2015). Being ectomycorrhizal, recognition of their host tree species and protection improve the yield of this mushroom substantially for commercial exploitation in view of food security and livelihood of native tribals.
